# Multiple molecular marker testing (p53, C-Ki-ras, c-erbB-2) improves estimation of prognosis in potentially curative resected non-small cell lung cancer

**DOI:** 10.1054/bjoc.2000.1287

**Published:** 2000-07-24

**Authors:** P M Schneider, H W Praeuer, O Stoeltzing, J Boehm, J Manning, R Metzger, U Fink, S Wegerer, A H Hoelscher, J A Roth

**Affiliations:** Departments of Visceral and Vascular Surgery, University of Cologne, Cologne, Germany; The Division of Surgery, Department of Thoracic SurgeryPathology, Technical University of Munich, Munich, Germany; Departments of Pathology, Thoracic and Cardiovascular Surgery, MD Anderson Cancer Center, Houston, Texas, USA

**Keywords:** lung cancer, tumour suppressor gene, p53, Ki-ras, c-erbB-2, prognosis

## Abstract

A prospective study was performed in patients with non-small cell lung cancer (NSCLC) to evaluate the prognostic importance of multiple molecular marker (p53, c-Ki-ras, c-erbB-2) testing. 103 patients with potentially curative resections (RO resection) for NSCLC in histopathological stages I–IIIA were included. SSCP analysis and DNA sequencing for p53 and c-Ki-ras genes were performed on paired tumour and normal lung tissue samples and immunohistochemistry (c-erbB-2) was done on frozen tissue sections with a specific anti-c-erbB-2 monoclonal antibody. 46/103 (44.6%) NSCLC showed p53 mutations and 17/103 (16.5%) c-Ki-ras mutations including 12/37 (32.4%) adenocarcinomas. Overexpression of c-erbB-2 (p185) was detected in 56/103 (54.4%) tumours. 24/103 (23.3%) NSCLC were negative for alterations in all 3 parameters (c-Ki-ras, p53 and p185) whereas 79/103 (76.7%) were positive for at least one of the 3 parameters. In a regression model including a multiple molecular marker parameter (negative for all 3 markers versus positive for at least one marker), histopathological stage (*P*< 0.00001), respectively the pT (*P*< 0.01) and pN (*P*< 0.00001) categories and the multiple molecular marker parameter (*P*< 0.01) were of significant prognostic importance. This study demonstrates that testing 3 molecular markers (c-Ki-ras, p53 and c-erbB-2) improves estimation of prognosis compared to single marker testing and appears to define low (82.6% ± 7.9% 5-year survival) and high risk (40.2% ± 5.5% 5-year survival) groups for treatment failure in potentially curative (RO) resected NSCLC. © 2000 Cancer Research Campaign


British Journal of Cancer (2000) **83**(4), 473–479
© 2000 Cancer Research Campaign
doi: 10.1054/ bjoc.2000.1287, available online at http://www.idealibrary.com on IDEAL®

### Multiple molecular marker testing (p53, C-Ki-ras, c-erbB-2) improves estimation of prognosis in potentially curative resected non-small cell lung cancer

**PM Schneider[1], HW Praeuer[3], O Stoeltzing[1], J Boehm[4], J Manning[5], R Metzger[1], U Fink[2], S Wegerer[2], AH Hoelscher[1] and JA Roth[6]**

[1]Departments of Visceral and Vascular Surgery, University of Cologne, Cologne, Germany; [2]The Division of Surgery, and [3]Department of Thoracic Surgery and [4]Pathology, Technical University of Munich, Munich, Germany; [5]Departments of Pathology and [6]Thoracic and Cardiovascular Surgery, MD Anderson Cancer Center, Houston, Texas, USA

**Summary** A prospective study was performed in patients with non-small cell lung cancer (NSCLC) to evaluate the prognostic importance of multiple molecular marker (p53, c-Ki-ras, c-erbB-2) testing. 103 patients with potentially curative resections (RO resection) for NSCLC in histopathological stages I–IIIA were included. SSCP analysis and DNA sequencing for p53 and c-Ki-ras genes were performed on paired tumour and normal lung tissue samples and immunohistochemistry (c-erbB-2) was done on frozen tissue sections with a specific anti-c-erbB-2 monoclonal antibody. 46/103 (44.6%) NSCLC showed p53 mutations and 17/103 (16.5%) c-Ki-ras mutations including 12/37 (32.4%) adenocarcinomas. Overexpression of c-erbB-2 (p185) was detected in 56/103 (54.4%) tumours. 24/103 (23.3%) NSCLC were negative for alterations in all 3 parameters (c-Ki-ras, p53 and p185) whereas 79/103 (76.7%) were positive for at least one of the 3 parameters. In a regression model including a multiple molecular marker parameter (negative for all 3 markers versus positive for at least one marker), histopathological stage ($P < 0.00001$), respectively the pT ($P < 0.01$) and pN ($P < 0.00001$) categories and the multiple molecular marker parameter ($P < 0.01$) were of significant prognostic importance. This study demonstrates that testing 3 molecular markers (c-Ki-ras, p53 and c-erbB-2) improves estimation of prognosis compared to single marker testing and appears to define low (82.6% ± 7.9% 5-year survival) and high risk (40.2% ± 5.5% 5-year survival) groups for treatment failure in potentially curative (RO) resected NSCLC. © 2000 Cancer Research Campaign

**Keywords:** lung cancer; tumour suppressor gene; p53; Ki-ras; c-erbB-2; prognosis

Lung cancer was the most common cause of cancer death in 1996 in the United States and Germany (Parker, 1996). The World Health Organization histologic classification of lung cancer (World Health Organization, 1982) describes 3 major subtypes of non-small cell lung cancer (NSCLC), squamous cell carcinoma (SCC), adenocarcinoma (AC) and large cell carcinoma (LC) which represent approximately 75% of all lung cancer cases. Radical surgery (RO resection) offers the only chance for cure in patients with NSCLC and survival probabilities are mainly dependent on tumour stage (Mountain, 1986; Buelzebruck et al, 1992).

A variety of molecular markers have been implicated both in the pathogenesis and prognosis of NSCLC (Gazdar, 1992; Minna, 1993; Johnson, 1995). Conflicting results, however, were reported in the literature and this might be due to small sample sizes, non-homogeneous populations (Simon and Altman, 1994) and the use of optimal cut-off values (Altman et al, 1994). Also, various techniques were employed without proper methodological evaluation (Press et al, 1994). In general, data on treatment quality control are lacking in molecular prognostic marker studies although they are essential for the interpretation of trial results (Holmes, 1994).

We first reported differential expression of the c-erbB-2 gene in small cell versus non-small cell lung cancer (Schneider et al, 1989) and shortly thereafter c-erbB-2 protein (p185) overexpression was reported to predict shortened survival in lung adenocarcinomas in a retrospective analysis (Kern et al, 1990). We therefore started a prospectice study in patients with curatively (RO category) resected NSCLC to conclusively evaluate the prognostic importance of increased expression of c-erbB-2 protein. In addition, the prognostic impact of mutations in the p53 tumor-suppressor gene (Chiba et al, 1990; Kishimoto et al, 1992; Mitsudomi et al, 1993; Carbone et al, 1994; Ryberg et al, 1994; Sauter et al, 1995) and c-Ki-ras gene (Rodenhuis and Slebos, 1992; Keohavong et al, 1996; Rosell et al, 1996) were evaluated since contradictory results were reported in the literature. The major purpose of this study was to evaluate if multiple molecular marker (c-Ki-ras, p53 and c-erbB-2) testing results in a better definition of low and high risk NSCLC patients for failure of standardized treatment.

*Received 16 November 1999*
*Revised 27 March 2000*
*Accepted 10 April 2000*

*Correspondence to:* PM Schneider MD, Assistant Professor of Surgery, Department of Visceral and Vascular Surgery, University of Cologne, 50931 Cologne, Germany. E-mail: Paul.Schneider@Medizin.Uni-Koeln.de

### PATIENTS AND METHODS

### Study design, demographic data and follow-up

From 136 patients consecutively operated for the clinical diagnosis of lung cancer, 103 patients fulfilled the following inclusion criteria and were prospectively evaluated: (I) NSCLC histology of

**Table 1** Survival in NSCLC based on clinical parameters

| Parameter | | *n* | 5-year survival (%) ± SD | Median survival (months) | CI 95% | *P* value |
|---|---|---|---|---|---|---|
| UICC stage | I | 49 | 73.1 ± 6.3 | n.r. | – | |
| | II | 23 | 55.7 ± 10.4 | n.r. | – | <0.0001 |
| | III A | 31 | 7.5 ± 5.3 | 18.8 ± 2.3 | 14.2; 23.3 | |
| pT | $pT_1$ | 24 | 70.8 ± 9.2 | n.r. | – | |
| | $pT_2$ | 65 | 48.6 ± 6.2 | 59.7 | – | <0.009 |
| | $pT_3$ | 14 | 21.4 ± 10.9 | 26.4 ± 7.3 | 12.0; 40.8 | |
| pN | $pN_0$ | 55 | 70.6 ± 6.1 | n.r. | – | |
| | $pN_1$ | 29 | 43.4 ± 9.4 | 45.4 ± 11.6 | 22.6; 68.2 | < 0.00001 |
| | $pN_2$ | 19 | 0 | 16.7 ± 2.9 | 10.9; 22.4 | |
| Histology | SCC | 49 | 54.5 ± 7.1 | n.r. | – | |
| | AC | 37 | 40.3 ± 8.0 | 45.4 ± 9.4 | 26.9; 63.9 | n.s. |
| | LC | 17 | 58.8 ± 11.9 | n.r. | – | |
| Grading | $G_1$ | 1 | (*n* = 1)* | – | – | |
| | $G_2$ | 23 | 51.8 ± 10.4 | n.r. | – | n.s. |
| | $G_3$ | 79 | 50.2 ± 5.6 | 63.9 | – | |

Abbreviations: n.r. (not reached); – (cannot be calculated); CI 95% (95% confidence interval); n.s. (not significant); *n* (number of patients)

the following WHO subtypes (World Health Organization, 1982): squamous cell carcinoma (SCC), adenocarcinoma (AC) or large cell carcinoma (LC); (II) complete tumour resection (RO category; Hermanek et al, 1993) by at least a lobectomy as minimal extent of resection; (III) systematic lymphadenectomy as quality control (Buelzebruck et al, 1992; Holmes, 1994); (IV): histopathological UICC stage I, II or IIIA; (V): postoperative radiotherapy for histopathological stage IIIA; (VI) no prior history of a malignant tumour or radiation and/or chemotherapy before sampling of the tissue specimes to be analysed; (VII) no perioperative 30-days lethality.

A total of 103 patients were included in the analysis. There were 79 male and 24 female patients with a median age of 63.5 years (min 34.5 y; max. 82.5 y). Tumours were radically removed by lobectomy (*n* = 67; 65%), bilobectomy (*n* = 13; 12.6%); pneumonectomy (*n* = 11; 10.7%) and extended pneumonectomy (*n* = 12; 11.7%) including mediastinal lymphadenectomy. Distribution of histopathological stages according to the UICC TNM Classification (I–IIIA), histological types according to the WHO classification (SCC, AC, LC) and histopathological grading of the primary tumour (G1–G3) is indicated (*n*) in Table 1. All patients in UICC Stage IIIA received postoperative radiotherapy.

Median follow-up was 86.8 months (min. 63.3; max. 105.2 months) and no patients was lost to follow-up. Patients were seen at 3 month intervals during the first postoperative year, every 6 months in the second and third year and once a year thereafter. Evaluation consisted of physical examination, biochemical profile, chest radiograph, CT scan of brain, chest and abdomen, abdominal ultrasound and technetium bone scan. Data on recurrences and cause of death were obtained for all patients.

### Tissue aquisition

Tissue for DNA analysis was obtained immediately after lung resection before starting mediastinal lymphadenectomy and was frozen in liquid nitrogen. Tissue was analysed from the following 2 locations: tumour and uninvolved lung tissue taken from the greatest distance to the tumour. Tumour tissue was selected from areas with at least 75% malignant cells.

### DNA analysis (c-Ki-ras and p53 gene)

DNA preparation was carried out with a DNA extraction kit according to the recommendations of the manufacturer (Stratagene Inc, LaJolla, CA). For PCR amplification, oligonucleotide primer pairs (MWG Biotech, Ebersberg, Germany) for exons 5–9 were chosen as reported (Imamura et al, 1993). The primers used for c-Ki-ras were: exon1: PKE1–1 (sense): 5′-ATGACTGAATA-TAAACTTGT-3′; PKE1–2 (antisense): 5′-CTCTATTGTTGGAT-CATATT-3′; exon2: PKE2–1 (sense): 5′-TTCCTACAGGAA-GCAAGTAGTA-3′; PKE2–2 (antisense): 5′-ACACAAAGAA-AGCCCTCCCCA-3′. The PCR, SSCP and DNA sequencing conditions were identical for p53 and c-Ki-ras gene analysis and have been previously reported with a sensitivity to detect 1 mutated cell in 10 non-mutated cells (Schneider et al, 1996).

### Immunohistochemistry (c-erbB-2 gene expression)

p185 immunostaining was performed on 6 µm cryostat sections. Frozen sections were air dried and fixed in acetone for 30 min followed by chloroform for 30 min both at 20°C. Sections were incubated for 30 min with a monoclonal IgG1 c-erbB-2 antibody (anti-c-erbB-2, Triton Biosciences, Alameda, CA, USA) diluted 1:500 that recognizes an epitope of the extracellular region of p185 and specifically immunoprecipitates p185 in Western blotting experiments (Iglehart et al, 1990). Staining was performed with the alkaline-phosphatase-antialkaline-phosphatase (APAAP)-complex (Dakopatts, Glostrup, Denmark) method as reported (Boenisch, 1989). Control sections for all probes were stained both after incubation with an isotype-specific monoclonal mouse antibody MOPC 21 (Sigma, St. Louis, MO, USA) and by omitting the primary antibody. Three frozen sections with paired tumour and normal lung tissue were processed for each patient probe. Scoring of immunohistochemical results was performed by two independent pathologists (JB and JM) who were blinded with regard to clinical data. The immunohistochemical staining was scored negative (grade 0) if membrane staining was absent, unequivocally positive but weak membrane staining (grade 1), moderate membrane staining (grade 2) and strong membrane staining (grade

 *© 2000 Cancer Research Campaign*

**Table 2** Survival in NSCLC based on molecular parameters

| Molecular parameter | | *n* | Subtype | 5-year survival (%) ± SD | Median survival (months) ± SD | CI 95% | *P* value |
|---|---|---|---|---|---|---|---|
| p185 | GRD 0 | 9 | ALL | 77.7 ± 13.8 | n.r. | – | |
| | GRD 1/2 | 38 | | 54.6 ± 8.1 | n.r. | – | |
| | GRD 3 | 56 | | 38.3 ± 7.2 | 39.3 ± 8.9 | 21.7; 56.9 | <0.05 |
| | GRD 0 | 0 | AC | Ø | Ø | Ø | |
| | GRD 1/2 | 6 | | 81.8 ± 16.4 | n.r. | – | |
| | GRD 3 | 31 | | 32.2 ± 8.4 | 35.8 ± 9.8 | 16.6; 55.0 | <0.01 |
| Ki-ras | NEG | 86 | ALL | 50.6 ± 5.4 | 63.9 | – | |
| | POS | 17 | | 47.0 ± 12.1 | 46.7 | – | n.s. |
| | NEG | 25 | AC | 38.7 ± 9.8 | 44.0 ± 19.7 | 5.9; 82.7 | |
| | POS | 12 | | 41.6 ± 14.2 | 45.4 ± 6.4 | 32.9; 58.0 | n.s. |
| p53 | NEG | 57 | ALL | 53.6 ± 6.6 | n.r. | – | |
| | POS | 46 | | 45.4 ± 7.3 | 43.1 ± 16.5 | 10.8; 75.5 | n.s. |
| | NEG | 20 | SCC | 64.8 ± 10.7 | n.r. | – | |
| | POS | 29 | | 48.0 ± 9.3 | 51.7 | – | n.s. |

Abbreviations: GRD (grading of immunohistochemical staining of p185: 0: no expression, 1/2: low-moderate expression, 2: overexpression); NEG (negative for c-Ki-ras or p53 mutations); POS (positive for c-Ki-ras or p53 mutations); n.r. (not reached); – (cannot be calculated); n.s. (not significant); CI 95% (95% confidence interval); Ø: no cases; *n* (number of patients)

3). There was no inter-observer variation in grade 0 and grade 3 staining, however, there was significant ($P < 0.01$) inter-observer variation in grade 1 and grade 2 staining by Kendall's test (Spurrier and Hewett, 1980). Therefore, the 2 groups (grade 1 and 2) were combined to the group of unequivocally positive low–moderate p185 expression.

### Statistical analysis

Kendall's analysis of concordance (Spurrier and Hewett 1980) was applied to test for inter-observer variation in evaluation of p185 immunohistochemical staining. Association of gene mutations or gene expression with clinico-pathological parameters were evaluated using the Chi-Square-Test. Correlations between groups were assessed by Spearman's correlation coefficient test. Survival was estimated according to Kaplan and Meier (1958). Univariate analysis was performed with the log-rank test (Mantel, 1966) and multivariate analysis with the Cox Proportional Hazard Regression Model (Cox, 1972) and significance was determined by $\chi^2$ analysis. The level of significance was set to $P < 0.05$.

### RESULTS

### Clinical data

Median and 5-year survival rates depending on various clinical variables are summarized in Table 1. Histopathological UICC tumour stage ($P < 0.0001$), pT category ($P < 0.009$), pN category ($P < 0.00001$) were of significant prognostic importance using the log rank test. Gender, age and grading of the primary tumour had no prognostic impact on survival.

### C-erbB-2 protein analysis

No expression of p185 was detected in 9/103 (8.7%), low-moderate expression in 38/103 (36.9%) and overexpression in 56/103 (54.4%) NSCLC samples (Figure 1). p185 overexpression was not limited but significantly ($P < 0.01$) associated with AC histology (31/56) compared to SCC (19/56) and LC (6/56). p185 overexpression was not associated with UICC histopathological tumour stage or grading of the primary tumour. NSCLC with p185 overexpression had a significantly reduced median and 5-y survival probability ($P < 0.05$) as shown in Table 2. Subgroup analysis based on the histological subtype revealed that p185 overexpression was significantly ($P < 0.01$) associated with reduced median and 5-year survival rates for patients with AC histology ($P < 0.01$) as shown in Table 2.

### c-Ki-ras analysis

SSCP analysis and DNA sequencing revealed the presence of c-Ki-ras mutations in 17/103 (16.5%) NSCLC. Mutations were significantly associated with AC histology ($P < 0.005$) and were present in 12/37 (32.4%) AC compared to 3/49 (6.1%) SCC and 2/15 (11.8%) LC. Detection of c-Ki-ras mutations were not associated with UICC histopathological tumour stage or grading of the primary tumour. 15/17 point mutations were found in codon 12, 2/17 in codon 13 and no mutation in codon 61. The predominant

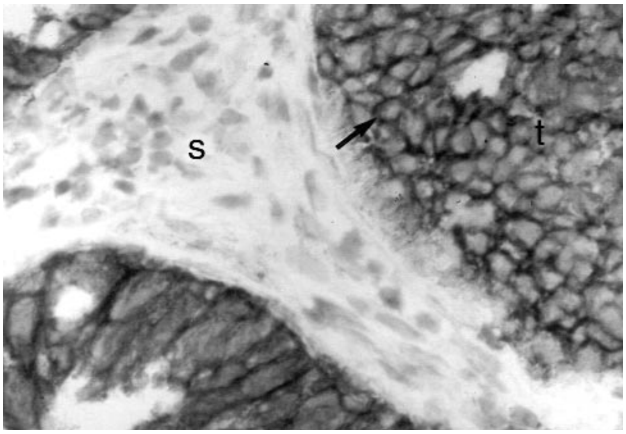

**Figure 1** Representative example of c-erbB-2 immunohistochemistry from a poorly differentiated adenocarcinoma (magnification 400×) showing overexpression of p185 with intensive red membrane staining (arrow) within the tumour (t). The interspersed stroma (s) is completely negative

**Table 3** Location and type of p53 mutation in NSCLC

| Location | Transversion | Transition | Deletion | Insertion | Total |
|---|---|---|---|---|---|
| Exon 5 | 15 | 9 | 1 | 1 | 26 (56.52%) |
| Exon 6 | 1 | 0 | 1 | 0 | 2 (4.35%) |
| Exon 7 | 7 | 1 | 2 | 0 | 10 (21.74%) |
| Exon 8 | 2 | 3 | 3 | 0 | 8 (17.39%) |
| Total | 25 (54.35%) | 13 (28.26%) | 7 (15.22%) | 1 (2.17%) | 46 (100%) |

Table 3 shows the distribution of exon locations and types of p53 mutations in 46 mutation-positive NSCLC. Mutations were most frequently located on exon 5 (56.52%) and were predominantly transversion-type mutations (54.35%). All mutations led to amino acid changes type of mutation was a G → T transversion in position 1 of codon 12 (glycine → cysteine; 8/17, 47%), with a G → T transversion in position 2 of codon 12 occurring in 11.8% of cases (glycine → valine; 2/17), a G → C transversion in position 2 in 11.8% (glycine → alanine; 2/17), a G → A transition in position 2 of codon 12 in 17.6% (glycine → aspartic acid; 3/17) and a G → T transversion in position 1 of codon 13 (glycine → cysteine; 2/17) in 11.8% of the cases.

Presence of c-Ki-ras mutations were not associated with reduced median and 5-year survival rates and subgroup analysis was statistically only possible for AC histology and did not show any significant impact on survival (Table 2).

### p53 analysis

DNA analysis of the p53 gene demonstrated the presence of mutations in 46/103 (44.7%) NSCLC and representative examples are shown in Figure 2. p53 mutations were significantly ($P < 0.01$) associated with the histological NSCLC subtype and were more frequent in SCC (59.2%) than AC (32.4%) or LC (29.4%). There was no association of p53 mutations with UICC stage and grading of the tumour. The distribution of the exon location and types of mutation are summarized in Table 3. Survival rates did not differ between NSCLC with and without p53 mutations (Table 2) and subgroup analysis demonstrated a tendency towards a reduced survival probability in SCC which was not statistically significant (Table 2).

### Multiple molecular parameter (c-Ki-ras, p53 and c-erbB-2) analysis

24/103 (23.3%) NSCLC did not show any alteration in one of the 3 markers (c-Ki-ras, p53 and p185) and are therefore multiple parameter marker negative. One parameter positivity was observed in 25/103 (24.3%) for p185, 20/103 (19.4%) for p53 and in 0/103 NSCLC for c-Ki-ras. In this analysis, the 2 subgroups with no expression of p185 and low-moderate p185 expression were combined to one group (no p185 overexpression) since there were no differences in survival probabilities by log rank test ($P$ = 0.5). Two molecular markers were positive in 8/103 (7.8%) for p185 and c-Ki-ras, 17/103 (16.5%) for p185 and p53 and 3/103 (2.9%) NSCLC for c-Ki-ras and p53 gene mutations. 6/103 (5.8%) showed abnormalities in all 3 parameters. There was no significant association of multiple marker positivity with UICC stage.

Survival data based on different combinations of the markers tested are shown in Table 4. Log-rank testing was statistically significant ($P < 0.001$) and demonstrated that tumours with single, double and triple marker positivity had a significantly worse prognosis when compared to 3 parameter negative tumours. Statistical subgroup analysis for the 8 combinations was not possible since 4 combinations (Ki-ras only, Ki-ras and p185, Ki-ras and p53, 3 marker positive) were infrequently detected (Table 4).

For further statistical analysis NSCLC were stratified as multiple parameter negative (no alteration in any of the 3 markers) or positive if one or more markers showed an alteration. Survival curves are shown in Figure 3 and demonstrate a significant difference ($P < 0.001$) with 5-year survival probabilities of 82.6% (± 7.9%) for negative cases compared to 40.2% (±5.5%) for positive cases.

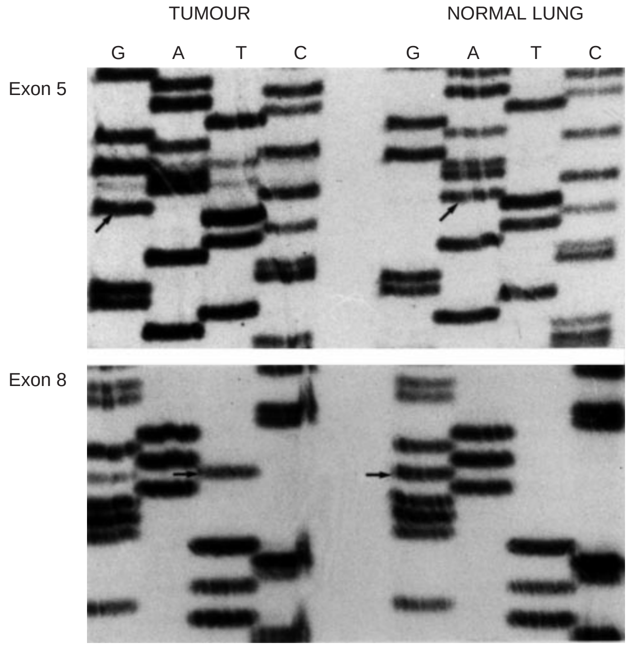

**Figure 2** Direct DNA sequencing analysis of 2 representative tumours and corresponding normal lung tissues. One tumour (top) shows an A → G transition-type missense mutation (arrows) in exon 5 and the other (bottom) a G → T transversion-type (arrows) missense mutation in exon 8

 © 2000 Cancer Research Campaign

**Table 4** Survival based on multiple molecular marker combinations

| Molecular parameter | No. cases | 5-year survival (%) ± SD | Median survival (months) ± SD | CI 95% |
|---|---|---|---|---|
| 3 marker negative | 24 | 82.6 ± 7.9 | n.r. | – |
| p185 only | 25 | 27.6 ± 9.2 | 20.8 ± 6.0 | 9.0–32.7 |
| p53 only | 20 | 28.2 ± 10.1 | 31.6 ± 16.7 | 0.0–64.5 |
| Ki-ras only | 0 | – | – | – |
| Ki-ras and p185 | 8 | 37.5 ± 17.1 | 35.8 ± 11.0 | 14.1–57.5 |
| Ki-ras and p53 | 3 | 66.6 ± 27.2 | n.r. | – |
| p185 and p53 | 17 | 56.2 ± 12.4 | n.r. | – |
| 3 marker positive | 6 | 50.0 ± 20.4 | 45.4 | – |

Abbreviations: n.r. (not reached) – (cannot be calculated); CI 95% (95% confidence interval)

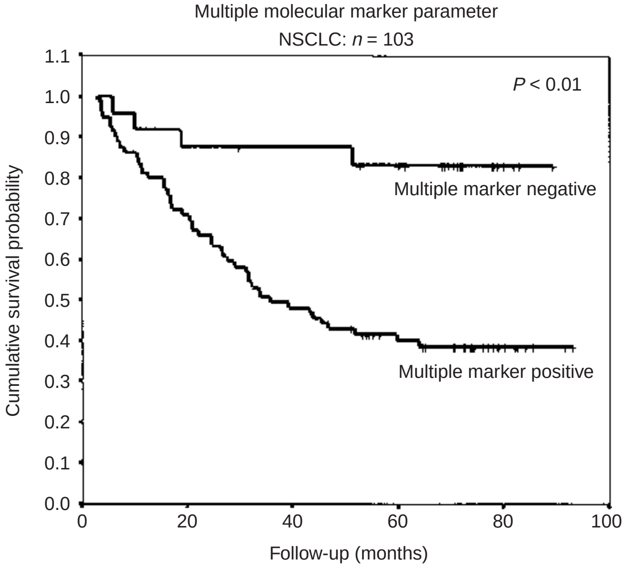

**Figure 3** Kaplan-Meier curves based on the multiple molecular marker parameter. NSCLC negative for the 3 markers c-Ki-ras, p53 and p185 (multiple marker negative) are compared to tumours positive for at least one of the 3 tested markers (multiple marker positive)

**Table 5** Cox-proportional hazard regression models

| Model | Parameter | *P* value | Odds ratio | CI 95% |
|---|---|---|---|---|
| A | Stage | 0.00001 | | |
| | I/II | 0.03 | 2.68 | 1.08–6.61 |
| | I/IIIA | 0.00001 | 9.17 | 4.36–19.26 |
| B | pT | 0.01 | | |
| | pT1/pT2 | 0.02 | 2.22 | 0.94–5.22 |
| | pT1/pT3 | 0.003 | 4.31 | 1.51–12.27 |
| | pN | 0.00001 | | |
| | pN0/pN1 | 0.03 | 1.91 | 1.08–5.14 |
| | pN0/pN2 | 0.00001 | 8.59 | 4.25–17.39 |
| C | Stage | 0.00001 | | |
| | I/II | 0.07 | 2.18 | 0.92–5.17 |
| | I/IIIA | 0.00001 | 6.19 | 3.07–12.49 |
| | Triple marker | 0.01 | 3.55 | 1.25–10.09 |
| D | pT | 0.01 | | |
| | pT1/pT2 | 0.03 | 2.38 | 1.05–5.41 |
| | pT1/pT3 | 0.004 | 4.22 | 1.56–11.39 |
| | pN | 0.00001 | | |
| | pN0/pN1 | 0.05 | 2.05 | 0.99–4.24 |
| | pN0/pN2 | 0.00001 | 7.71 | 3.80–15.62 |
| | Triple marker | 0.01 | 3.76 | 1.34–10.59 |

Abbreviations: CI 95%: 95% confidence interval for odds ratio. Parameter section: e.g. stage I/II means stage I compared to stage II

### Multivariate analysis

Four logistic regression models were tested. Model A included parameters gender, age, histological type, UICC tumour stage, grading of the primary tumour, p185 expression, p53 mutation and c-Ki-ras mutation and model B the pT and pN categories instead of histopathological tumour stage. Only UICC tumour stage ($P < 0.00001$) in model A respectively the pT ($P < 0.01$) and pN ($P < 0.00001$) categories in model B were of independent prognostic importance. In models C and D we used the multiple molecular parameter instead of p185, c-Ki-ras and p53. UICC tumour stage ($P < 0.00001$) in model C, the pT ($P < 0.01$) and pN ($P < 0.00001$) categories in model D and the multiple molecular parameter ($P > 0.01$) in models C and D were of significant independent prognostic importance. Table 5 shows the statistically significant parameters in the 4 regression models.

### DISCUSSION

We designed this prospective study to test the hypothesis that p185 overexpression, c-Ki-ras mutations and p53 mutations by themselves or in combination are of prognostic importance in patients with curatively (UICC RO) resected NSCLC. Our treatment results for curatively (UICC RO) resected NSCLC based on various parameters (UICC tumour stage, pT-category, pN-category, grading of the primary tumour and histological subtype) as shown in Table 1 are in good agreement with the large reference series in the literature (Buelzebruck et al, 1992; Mountain, 1986; Naruke et al, 1988).

The major problem in comparing studies of p185 expression in NSCLC is the enormous variation in frequencies of NSCLC tumours scored positive for p185. Overerexpression of p185 were reported in 28–80% AC (Kern et al, 1990; Bongiorno et al, 1994; Tateishi et al, 1991; Shi et al, 1992; Pfeiffer et al, 1996), 2–45% SCC (Kern et al, 1990; Pfeiffer et al, 1996; Shi et al, 1992; Tateishi, 1992; Volm et al, 1992) and 0–20% LC (Kern et al, 1990; Pfeiffer et al, 1996). Some of these studies used paraffin embedded slides (Kern et al, 1990; Tateishi, 1992; Volm et al, 1992) or applied p185 antiserum (Kern et al, 1990; Tateishi et al, 1991; Pfeiffer et al, 1996) for immunohistochemistry.

Chiu et al (1994) could convincingly demonstrate in a controlled comparative analysis of paraffin-embedded slides and cryostat sections that rates of p185 overexpression were approximately doubled in cryostat sections. Press et al (1994) tested

© 2000 Cancer Research Campaign 

7 polyclonal and 21 monoclonal anti-c-erbB-2 antibodies and demonstrated variations in positive membrane staining between 2–30% as well as significant differences in sensitivity and specificity. As a consequence, different results were reported concerning the impact of c-erbB-2 protein overexpression on survival in NSCLC. Kern et al (1990) and Tateishi et al (1991) reported significantly reduced survival rates for AC over-expressing p185. Both studies are retrospective analyses using paraffin-embedded slides and polyclonal antiserum. Careful review of the data presented by Kern et al (1990) reveals that their stage IIIA patients had a better prognosis than the patients in stage II and that the pN-category was not of significant prognostic importance ($P$ = 0.95). Pfeiffer et al (1996) did not report differences in survival in AC overexpressing p185 however, the staining frequency in AC was only 26%. In our study, p185 overexpression is of significant prognostic importance for AC histology ($P < 0.01$) by univariate analysis but not of independent prognostic importance by multivariate analysis.

Presence of c-Ki-ras mutations were reported in 15.7–36% (Kern, 1994; Keohavong et al, 1996; Mitsudomi et al, 1991; Rosell et al, 1996; Slebos et al, 1990; Sugio et al, 1992) of AC NSCLC which is consistent with our result of 32%. Slebos et al (1990) and Mitsudomi et al (1991) reported significant differences in survival if c-Ki-ras mutations were present in AC NSCLC. Kern and colleagues (1994) could not demonstrate significant impact on survival in 16/44 AC lung cancer patients with c-Ki-ras mutations however demonstrated an independent negative prognostic effect by multivariate analysis if c-Ki-ras mutations and p185 overexpression was present in AC ($P < 0.004$). In our study c- Ki-ras gene mutations were not of prognostic significance by univariate and multivariate analysis for patients with curatively resected AC NSCLC which is consistent with the more recently published reports (Keohavong et al, 1996; Rosell et al, 1996).

Mutations in the p53 tumour-suppressor gene were reported in 32%–52% NSCLC (Chiba et al, 1990; Horio et al, 1993; Kishimoto et al, 1992; Mitsudomi et al, 1993; Carbone et al, 1994; Ryberg et al, 1994; Sauter et al, 1995) and reported results are within the range of the 45% detected in our series. We did not find a significant prognostic impact on survival for all NSCLC if p53 mutations were present by both univariate or multivariate analysis. Horio et al (1993) reported significant worse survival data for 35/70 (49%) p53 mutation-positive NSCLC with potentially curative resections ($P < 0.014$) including subgroup analysis for stages I and II tumours ($P < 0.016$) and an independent impact on survival by multivariate analysis ($P < 0.013$). Mitsudomi and colleagues (1993) also showed a significant negative prognostic impact of p53 mutations ($P < 0.01$) but subgroup analysis revealed significant prognostic differences for advanced UICC stages IIIA to IV ($P < 0.009$) and not for early stages I and II ($P$ = 0.28). On the contrary, Top et al (1995) who evaluated 54 patients with resected NSCLC did see a better prognosis in p53 mutation-positive tumours. The lung cancer study group 871 (Carbone et al, 1994) failed to show a prognostic impact in curatively resected NSCLC carrying p53 mutations ($P$ = 0.62) but detected a weak negative survival correlation with positive p53 immunostaining ($P < 0.05$).

One of the critical questions is the evaluation of the interrelationships between factors reported to be of prognostic importance. Only few studies have already addressed this question and Kern and colleagues (1994) examined the interrelationship of c-Ki-ras mutations and p185 overexpression and found a significantly worse prognosis for lung adenocarcinomas harbouring c-Ki-ras mutations and p185 overexpression. Volm et al (1992) examined the expression at the protein level of c-erbB-1, c-erbB-2, c-fos, c-myc and p53 using immunohistochemistry and found that c-erbB-1 and c-fos expression may serve as prognostic factors for the aggressiveness of SCC and response to chemotherapy.

Our study is the first prospective controlled study examining the three most frequently examined and controversially discussed prognostic parameters in RO resected NSCLC: p53 mutations, c-Ki-ras mutations and c-erbB-2 protein expression. Our data convincingly demonstrate that 3 parameter negative tumours have a highly significant better prognosis than tumours testing positive for at least one molecular marker.

The major conclusion of this study is that multiple molecular marker testing is necessary to detect an independent prognostic impact on survival and is therefore superior to single marker testing if p53 and c-Ki-ras mutations and c-erbB-2 overexpression are evaluated. Based on multiple molecular marker testing 2 groups of curatively resected NSCLC could be defined: a low-risk group (multiple molecular marker negative) and a high-risk group (multiple molecular marker positive) for failure of standardized treatment.

This information might be most valuable in the planning of pre- or postresection treatment strategies in future studies.

### ACKNOWLEDGEMENT

The authors wish to thank E Bollschweiler, MD, PhD, Statistics, EDV and Documentation Unit of the Department of Visceral and Vascular Surgery, University of Cologne, Germany for assisting with the statistical analysis.

### REFERENCES

Altman DG, Lausen B, Sauerbrei W and Schumacher M (1994) Dangers of using 'optimal' cutpoints in the evaluation of prognostic factors. *J Natl Cancer Inst* **86:** 829–835

Boenisch T (1989) Staining methods, controls, background. In: *Handbook Immunochemical Staining Methods*. Dako Carpinteria, pp 13–23

Bongiorno PF, Whyte RI, Lesser EJ, Moore JH, Orringer MB and Beer DG (1994) Alterations of K-ras, p53, and erbB-2/neu in human lung adenocarcinomas. *J Thorac Cardiovasc Surg* **107:** 590–595

Buelzebruck H, Bopp R, Drings P, Bauer E, Krysa S, Probst G, van Kaick G, Muller KM and Vogt-Moykopf I (1992) New aspects in the staging of lung cancer. Prospective validation of the International Union Against Cancer TNM classification. *Cancer* **70:** 1102–1110

Carbone DP, Mitsudomi T, Chiba I, Piantadosi S, Rusch V, Nowak JA, McIntire D, Slamon D, Gazdar A and Minna J (1994) p53 immunostaining positivity is associated with reduced survival and is imperfectly correlated with gene mutations in resected non-small cell lung cancer: a preliminary report of Lesg 871. *Chest* **106:** 377–381

Chiba I, Takahashi T, Nau MM, D DA, Curiel DT, Mitsudomi T, Buchhagen DL, Carbone D, Piantadosi S, Koga H, and et al. (1990) Mutations in the p53 gene are frequent in primary, resected non-small cell lung cancer. *Lung Cancer Study Group, Oncogene* **5:** 1603–1610.

Chiu KY, Loke SL and Ho FC (1994) Immunohistochemical demonstration of c-erbB-2 oncoprotein in gastric adenocarcinoma: comparison of cryostat and paraffin wax sections and effect of fixation. *J Clin Pathol* **47:** 117–121

Cox DR (1972) Regression models and life-tables (with discussion). *J R Stat Soc B* **34:** 187–220

Gazdar AF (1992) The molecular biology of lung cancer. *Tohoku J Exp Med* **168:** 239–245

 © 2000 Cancer Research Campaign

Hermanek P, Henson DE, Hutter RVP, and Sobin LH (1993) UICC TNM Supplement. Springer Verlag, Berlin, Heidelberg, New York 9–14

Holmes EC (1994) General principles of surgery quality control. *Chest* **106:** 334–336

Horio Y, Takahashi T, Kuroishi T, Hibi K, Suyama M, Niimi T, Shimokata K, Yamakawa K, Nakamura Y, Ueda R and et al. (1993) Prognostic significance of p53 mutations and 3p deletions in primary resected non-small cell lung cancer. *Cancer Res* **53:** 1–4

Iglehart JD, Kraus MH, Langton BC, Huper G, Kerns BJ and Marks JR (1990) Increased c-erbB-2 gene copies and expression in multiple stages of breast cancer. *Cancer Res* **50:** 6701–6707

Imamura J, Bartram CR, Berthold F, Harms D, Nakamura H and Koeffler HP (1993) Mutation of the p53 gene in neuroblastoma and its relationship with N-myc amplification. *Cancer Res* **53:** 4053–4058

Johnson BE (1995) Biologic and molecular prognostic factors-impact on treatment of patients with non-small cell lung cancer. *Chest* **107:** 287–290

Kaplan EL and Meier P (1958) Nonparametric estimation from incomplete observations. *J Am Stat Assoc* **53:** 187–220

Keohavong P, DeMichele MA, Melacrinos AC, Landreneau RJ, Weyant RJ and Siegfried JM (1996) Detection of K-ras mutations in lung carcinomas: relationship to prognosis. *Clin Cancer Res* **2:** 411–418

Kern JA, Schwartz DA, Nordberg JE, Weiner DB, Greene MI, Torney L and Robinson RA (1990) p185neu expression in human lung adenocarcinomas predicts shortened survival. *Cancer Res* **50:** 5184–5187

Kern JA, Slebos RJ, Top B, Rodenhuis S, Lager D, Robinson RA, Weiner D and Schwartz DA (1994) C-erbB-2 expression and codon 12 K-ras mutations both predict shortened survival for patients with pulmonary adenocarcinomas. *J Clin Invest* **93:** 516–520

Kishimoto Y, Murakami Y, Shiraishi M, Hayashi K and Sekiya T (1992) Aberrations of the p53 tumor suppressor gene in human non-small cell carcinomas of the lung. *Cancer Res* **52:** 4799–804

Mantel N (1966) Evaluation of survival data and two new rank order statistics arising in its consideration. *Chemother Rep* **50:** 163–170

Minna JD (1993) The molecular biology of lung cancer pathogenesis. *Chest* **103:** 449–456

Mitsudomi T, Steinberg SM, Oie HK, Mulshine JL, Phelps R, Viallet J, Pass H, Minna JD and Gazdar AF (1991) ras gene mutations in non-small cell lung cancers are associated with shortened survival irrespective of treatment intent. *Cancer Res* **51:** 4999–5002

Mitsudomi T, Oyama T, Kusano T, Osaki T, Nakanishi R and Shirakusa T (1993) Mutations of the p53 gene as a predictor of poor prognosis in patients with non-small-cell lung cancer. *J Natl Cancer Inst* **85:** 2018–2023

Mountain CF (1986) A new international staging system for lung cancer. *Chest* **89:** 225–233

Mountain CF (1990) Expanded possibilities for surgical treatment of lung cancer. Survival in stage IIIa disease. *Chest* **97:** 1045–1051

Naruke T, Goya T, Tsuchiya R and Suemasu K (1988) Prognosis and survival in resected lung carcinoma based on the new international staging system [published erratum appears in J Thorac Cardiovasc Surg 1989 Mar; 97(3):350]. *J Thorac Cardiovasc Surg* **96:** 440–447

Parker SL, Tong T, Bolden S and Wingo PA (1996) Cancer statistics, 1996. *CA Cancer J Clin* **46:** 5–27

Pfeiffer P, Clausen PP andersen K and Rose C (1996) Lack of prognostic significance of epidermal growth factor receptor and the oncoprotein p185HER-2 in patients with systemically untreated non-small-cell lung cancer: an immunohistochemical study on cryosections. *Br J Cancer* **74:** 86–91

Press MF, Hung G, Godolphin W and Slamon DJ (1994) Sensitivity of HER-2/neu antibodies in archival tissue samples: potential source of error in immunohistochemical studies of oncogene expression. *Cancer Res* **54:** 2771–2777

Quinlan DC, Davidson AG, Summers CL, Warden HE and Doshi HM (1992) Accumulation of p53 protein correlates with a poor prognosis in human lung cancer. *Cancer Res* **52:** 4828–4231

Rodenhuis S and Slebos RJ (1992) Clinical significance of ras oncogene activation in human lung cancer. *Cancer Res* **52:** 2665–2669

Rosell R, Gomez-Codina J, Camps C, Maestre J, Padille J, Canto A, Mate JL, Li S, Roig J, Olazabal A and et al. (1994) A randomized trial comparing preoperative chemotherapy plus surgery with surgery alone in patients with non-small-cell lung cancer. *N Engl J Med* **330:** 153–8

Rosell R, Monzo M, Pifarre A, Ariza A, Sanchez JJ, Moreno I, Maurel J, Lopez MP, Abad A and De Anta JM (1996) Molecular staging of non-small cell lung cancer according to K-ras genotypes. *Clin Cancer Res* **2:** 1083–1096

Ryberg D, Kure E, Lystad S, Skaug V, Stangeland L, Mercy I, Borresen AL and Haugen A (1994) p53 mutations in lung tumors: relationship to putative susceptibility markers for cancer. *Cancer Res* **54:** 1551–1555

Sauter ER, Gwin JL, Mandel J and Keller SM (1995) p53 and disease progression in patients with non-small cell lung cancer. *Surg Oncol* **4:** 157–161

Schneider PM, Hung MC, Chiocca SM, Manning J, Zhao XY, Fang K and Roth JA (1989) Differential expression of the c-erbB-2 gene in human small cell and non-small cell lung cancer. *Cancer Res* **49:** 4968–4971

Schneider PM, Casson AG, Levin B, Garewal HS, Hoelscher AH, Becker K, Dittler HJ, Cleary KR, Troster M, Siewert JR and Roth JA (1996) Mutations of p53 in Barrett's esophagus and Barrett's cancer: a prospective study of ninety-eight cases. *J Thorac Cardiovasc Surg* **111:** 323–331; discussion 331–333

Shi D, He G, Cao S, Pan W, Zhang HZ, Yu D and Hung MC (1992) Overexpression of the c-erbB-2/neu-encoded p185 protein in primary lung cancer. *Mol Carcing* **5:** 213–218

Simon R and Altman DG (1994) Statistical aspects of prognostic factor studies in oncology. *Br J Cancer* **69:** 979–85

Slebos RJ, Kibbelaar RE, Dalesio O, Kooistra A, Stam J, Meijer CJ, Wagenaar SS, Vanderschueren RG, van Zandwijk N, Mooi WJ and et al. (1990) K-ras oncogene activation as a prognostic marker in adenocarcinoma of the lung. *N Engl J Med* **323:** 561–565

Spurrier JD and Hewett JE (1980) Two-stage test of independence using Kendall's statistic. *Biometrics* **36:** 517–522

Sugio K, Ishida T, Yokoyama H, Inoue T, Sugimachi K and Sasazuki T (1992) ras gene mutations as a prognostic marker in adenocarcinoma of the human lung without lymph node metastasis. *Cancer Res* **52:** 2903–2906

Tateishi M, Ishida T, Mitsudomi T, Kaneko S and Sugimachi K (1991) Prognostic value of c-erbB-2 protein expression in human lung adenocarcinoma and squamous cell carcinoma. *Eur J Cancer* **27:** 1372–1375

Top B, Mooi WJ, Klaver SG, Boerrigter L, Wisman P, Elbers HR, Visser S and Rodenhuis S (1995) Comparative analysis of p53 gene mutations and protein accumulation in human non-small-cell lung cancer. *Int J Cancer* **64:** 83–91

Volm M, Efferth T and Mattern J (1992) Oncoprotein (c-myc, c-erbB1, c-erbB2, c-fos) and suppressor gene product (p53) expression in squamous cell carcinomas of the lung. Clinical and biological correlations. *Anticancer Res* **12:** 11–20

World Health Organization (1982) The World Health Organization histologic typing of lung tumors. *Am J Clin Pathol* **77:** 123–136

© 2000 Cancer Research Campaign 
